# Prevention of vertical transmission of hepatitis B: A retrospective review of a 5‐year maternal–infant cohort in London

**DOI:** 10.1111/jpc.16609

**Published:** 2024-07-02

**Authors:** Elizabeth O'Mahony, Sophie Raghunanan, Ashley Brown, Caroline Foster

**Affiliations:** ^1^ Oxford Vaccine Group, Department of Paediatrics University of Oxford University – Medical Sciences Oxford United Kingdom; ^2^ Department of Paediatric Infectious Diseases Imperial College Healthcare NHS Trust London United Kingdom; ^3^ Department of Hepatology Imperial College London, Imperial College NIHR BRC London United Kingdom

**Keywords:** hepatitis B, hepatitis B birth dose vaccination, hepatitis B immunoglobulin (HBIG), tenofovir disoproxil fumerate (TDF), vertical transmission

## Abstract

**Aims:**

The World Health Organization (WHO) estimates that 3.5% of the population live with hepatitis B virus (HBV); migrants to Europe are disproportionately affected. UK birth dose HBV vaccination is limited to infants born to those living with HBV (LWHBV). High‐risk infants (high maternal infectivity, low birthweight) also receive HBV immunoglobulin (HBIG). The Family Hepatitis Clinic follows infants and those LWHBV working towards WHO goals of combating viral hepatitis by 2030.

**Methods:**

A trust‐wide electronic note review of outcomes for infants born to those LWHBV (2016–2020).

**Results:**

Two hundred and eighty‐three infants, 134 (47%) females, born to those LWHBV were referred. Two hundred and thirty‐one (82%) attended follow‐up with a vertical transmission rate of 0%. Twenty (7%) individuals LWHBV received tenofovir disoproxil fumerate in pregnancy; median viral load (VL) at initiation 125 416 376 DNA IU/mL, one having birth VL. Twenty‐eight (10%) infants were stratified as high risk and all received HBIG and birth dose vaccination with 9 (32%) subsequently lost to follow‐up, compared to 48 (19%) low‐risk infants. 267/283 (94%) had birth dose vaccination documented and 206/283 (73%) received at least four vaccine doses. 215/283 (76%) infants had serology by 24 months; 17 (6%) with suboptimal vaccine responses: hepatitis B surface antibody <100 IU/mL. Serology before 18 months resulted in higher rates of maternal hepatitis B core antibody detection (15% vs. 3%).

**Conclusion:**

Prevention of vertical transmission of HBV was universal in those attending, although high‐risk infants were more likely lost to follow up. HBV post‐vaccine serological protection was comparable with national data from 2021 (77% >4 doses, 77% HBsAb >100).

## What is already known on this topic

1. Hepatitis B remains a global problem and in the UK underserved populations are disproportionately affected.
2. Effective interventions to reduce vertical transmission are in place globally, including in the UK, but with insufficient coverage to eliminate viral hepatitis.
3. Barriers, varying across the globe, prevent universal implementation of interventions required to meet the WHO 2030 goals.

## What this paper adds

1. Interventions to reduce HBV transmission are highly effective, but high‐risk infants are more likely to be lost to follow up without confirmatory serological testing of absence of infection and vaccination efficacy.
2. Testing hepatitis B core antibody in infants prior to 18 months increases the chance of positive results (indicative of maternal antibody transfer) and increases the burden of sampling and appointments.
3. Hepatitis B viral load monitoring, particularly around time of delivery, could improve risk stratification and optimise HBIG use in birth plans.

## Background

### Epidemiology and guidance: Global

The World Health Organization (WHO) estimate that 296 million people live with hepatitis B virus (HBV), 3.5% of the global population; the highest disease burden in the Western Pacific and African WHO regions.^1^ The 2022–2030 Global Health Sector Strategies aims to reduce new HIV and viral hepatitis cases from 4.5 million to less than 500 000 annually, and improve coverage of timely birth dose vaccination to 90% by 2030.^2^ These goals require high‐quality strategies, including routine screening for chronic hepatitis B infection in pregnancy, adoption of hepatitis B birth dose, infant vaccination and provision of antivirals in pregnancy where appropriate. Without intervention, there is a high risk of vertical transmission, especially where there is seropositivity for hepatitis B e‐antigen or viral loads over 200 000 IU/mL,^3^ and therefore is a significant area to address to globally reduce cases of hepatitis B.

Birth dose vaccination of Hepatitis B has been a WHO recommendation since 2009^4^; however, it remains far from universally available – with an estimated 50% coverage in 2020.^2^ There are numerous barriers to implementation: lack of education, limited availability of appointments, cold chain costs and staffing capacity.^5^ This highlights the importance of strategies that tackle these barriers and engage communities to work towards viral hepatitis elimination – a crucial strand in the WHO 2022–2030 strategies reiterated by the latest WHO guidance.^3^ Although a valuable tool in preventing transmission, birth dose alone is not sufficient to achieve elimination and whilst reducing infants' infection rate,^6^ studies have shown that up to 6% of those receiving vaccination still acquired hepatitis B.^7^ This highlights the importance of routine antenatal screening and use of maternal antivirals where appropriate.

### Epidemiology and guidance: United Kingdom

With an increasing globally mobile population, strategies must be in place across the world to ensure robust coverage for migrant populations. In the UK, Public Health England (PHE) data give an annual incidence for HBV of 0.68 per 100 000 in 2018^8^; the United Kingdom Health Security Agency (UKHSA) give an indirect estimate of 0.45% of the population living with chronic HBV infection.^9^ The UKHSA's 2023 Hepatitis report suggests over 95% of this disease burden was in migrant populations who acquired the infection in endemic countries prior to migration.^10^

A review of current evidence for preventing vertical transmission of HBV identified universal antenatal screening, selective use of antivirals in pregnancy, targeted birth dose vaccination, routine infant immunisation and hepatitis B immunoglobulin (HBIG) where indicated, as key strategies in use.^11^ Barriers to these interventions are an important consideration: a national audit of those living with HBV (LWHBV) accessing antenatal care in 2014 showed 25% had arrived in the UK <2 years ago, 39% had basic English or less, and 18% reported missed specialist appointments.^12^ These are all factors that affect health‐care access and concordance, so may reduce coverage of these interventions.

**Fig. 1:**
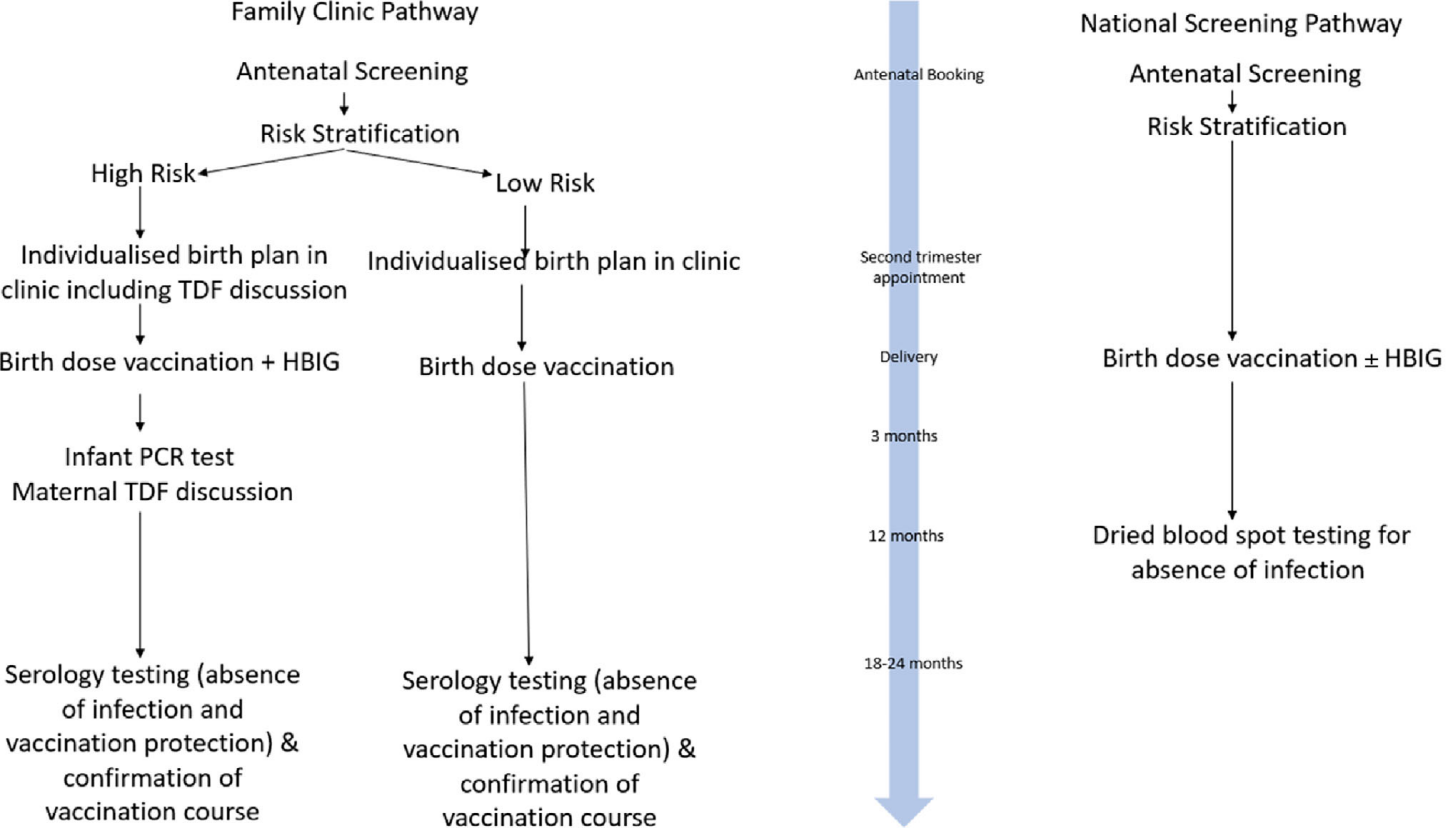
Flow diagram of HBV stratification and prevention pathway within the Family Clinic for both high‐ and low‐risk infants and the national screening service. HBIG, HBV immunoglobulin; HBV, hepatitis B virus; TDF, tenofovir disoproxil fumarate.

Universal antenatal HBV screening has been routine in the UK since 2000, with all pregnant people being offered screening with infectivity markers where required. Data are reported to the national Integrated Screening Outcomes Surveillance Service; monitoring obstetric and paediatric outcomes for infants exposed to HIV, HBV and syphilis perinatally.^13^ Pregnant people LWHBV are risk stratified based on the UKHSA High‐Risk Criteria (Table 1) – allowing comprehensive maternal and neonatal birth plans to be in place prior to delivery with recommendation for HBIG for high‐risk infants (Fig. 1).

**Table 1:** UKHSA high‐risk criteria

UKHSA high‐risk criteria^16^ HBsAg positive AND one of the following: HBeAg positive OR anti‐HBe negative OR e markers not available Acute hepatitis B in pregnancy Infant birthweight <1500 g HBV DNA ≥1 × 10^6^ iu/mL in any antenatal sample from this pregnancy

HBeAg, hepatitis B e‐antigen; HBsAg, hepatitis B surface antigen; UKHSA, United Kingdom Health Security Agency.

HBV was added to the routine infant immunisation UK schedule in 2017,^14^ as a three‐dose schedule using the new hexavalent DTaP/IPV/Hib/HepB (diphtheria, tetanus, pertussis, polio, Hib and hepatitis B) combination vaccine. This replaced the pentavalent vaccine in the standing immunisation schedule at 8, 12 and 16 weeks following revaluation by the Joint Committee of Vaccination and Immunisation.^15^ The neonatal selective schedule for hepatitis B vaccination prior to this introduction for HBV‐exposed infants recommended four doses: at delivery, 1 month, 2 months and a booster at 1 year. The decision to include HBV within the routine DTP‐containing hexavalent vaccine, meant the selective neonatal schedule also changed, in order to maintain the post‐exposure prophylaxis doses required at birth and 1 month, but also ensure no DTP‐containing vaccines would be missed. Therefore, the accompanying recommendation for infants born to those LWHBV was for six doses: monovalent at birth and 1 month, hexavalent at 2, 3 and 4 months and the monovalent booster at 1 year.

Within the UK national surveillance screening program mentioned above (issoss-online.org)^13^ infants born to those LWHBV are offered dried blood spot testing through primary care to ascertain HBV infection status at 1 year of age. This is offered as a heel prick blood test and arranged through the registered GP service.

### The family hepatitis clinic service

The Family Hepatitis Clinic (London, UK) offers an enhanced service for all pregnant people LWHBV who have antenatal care at the trust. They are offered screening for HBV at routine antenatal booking and any positive results are referred to a specialist midwife. Joint antenatal appointments with an adult hepatologist during the second trimester create a maternal and neonatal plan with interventions based on risk stratification as per the UKHSA criteria.^16^ Those with high HBV viral loads are offered tenofovir disoproxil fumarate (TDF) during the third trimester.^11^

The Family Hepatitis Clinic offers subsequent follow‐up of the infants and their mothers in a joint outpatient service staffed by adult hepatology and paediatric infectious diseases. Mothers and their infants are seen at 12–24 months to confirm vaccination course, check HBV serology to ensure a protective response to vaccination (hepatitis B surface antibody (HBsAb)) and confirm the absence of infection (hepatitis B surface antigen (HBsAg)). High‐risk infants are offered an additional appointment at 3 months of age with an HBV DNA PCR to ascertain infection status and for maternal discussion around TDF continuation or cessation. Figure 1 illustrates the pathway within the Family Hepatitis Clinic and the national screening service. Combining the infant and maternal clinics reduces appointment burden, an important factor affecting attendance, especially in a population who may disengage with care^17^; this mechanism facilitates subsequent post‐partum re‐integration into adult hepatology clinics promoting improved follow‐up for those LWHBV. In addition, the clinic will also review the HBV status of any siblings in the family, providing testing and vaccination if not under follow‐up or unsure of care history.

## Methods

Electronic notes of all maternal–infant pairs registered through the Family Clinic database were reviewed retrospectively. Infants were included if they were born between 1 January 2016 and 31 December 2020 to allow at least 30 months of follow‐up post‐delivery to maximise ascertainment of infant infection status due to rescheduling of appointments and delays due to the COVID‐19 pandemic. Infant data collected included demographics, HBV immunisation record and serology testing. Risk stratification and rationale, along with HIBIG administration were also documented. The associated maternal notes were reviewed where possible for demographics, HBV serology, HBe antibody status, viral load by HBV DNA PCR, co‐infection with hepatitis Delta (HDV), HIV and syphilis and prescription of TDF.

Vaccination records were collated and recorded with number of doses given. As the data collection period spans the change in HBV vaccine schedule from four to six, when estimating vaccine coverage and schedule adherence, four doses have been used as a bar for completing a vaccination course.

Data were anonymised and collated in a protected digital spreadsheet with median and interquartile ranges (IQRs) summarising non‐normally distributed continuous variables and numbers and percentages summarising categorical variables.

The study was registered with the local trust Audit Committee, categorised as an audit of routinely collected clinical data to quality assess current clinical practice, and hence research ethics were not required.

## Results

From 2016 to the end of 2020, 283 infants born to those LWHBV were registered; 134 (47%) were female, 202 (71%) were born to first‐generation migrants with 77 (27%) having unrecorded maternal country of birth and 1 (0.3%) being a UK born second‐generation migrant.

### Risk stratification

Risk stratification based on UKHSA criteria assigned 28 (10%) mother‐infant pairs to the high‐risk pathway; 23 (82%) due to maternal factors, 2 (7%) due to low birthweight <1500 g, and 1 (3.5%) due to HDV co‐infection (see table 2).

**Table 2:** Characteristics of mother–infant pairs

Demographics			*n* (%)
	Total cohort		283
Maternal demographics	Maternal age (*n* = 262)	Median [IQR]	33 [26–40]
	Maternal country of birth (*n* = 203)	Born abroad	202 (99.5%)
		UK	1 (0.5%)
	Maternal country of birth by WHO Global Region (*n* = 185)	African	32 (17%)
		Eastern Mediterranean	43 (23%)
		European	67 (36%)
		Region of the Americas	2 (1%)
		South East Asia	15 (8%)
		Western Pacific	26 (14%)
Maternal HBV markers	Serology (*n* = 263)	E‐antigen positive recorded	10 (4%)
	Maternal viral titres at booking (*n* = 233)	<10 iu/mL	16 (7%)
		11–100 iu/mL	74 (32%)
		101–1000 iu/mL	72 (31%)
		1000–1 000 000 iu/mL	58 (25%)
		>1 000 000 iu/mL	12 (5%)
	Maternal co‐infection present (*n* = 253)	Hepatitis C co‐infection	0
		Hepatitis D co‐infection	3 (1%)
		HIV co‐infection	2 (0.8%)
		Syphilis co‐infection	3 (1%)
Maternal HBV therapy	Maternal tenofovir (*n* = 20)	Pre‐conception tenofovir continued through pregnancy	8 (40%)
		Prescribed tenofovir for third trimester	12 (60%)
		Viral suppression demonstrated (<1000 iu/mL)	10 (50%)
Infant demographics (*n* = 283)	Gender	Male	149 (53%)
	Birthweight	<1.5 kg	2 (0.7%)
	Risk stratification	High risk as per UKHSA criteria	28 (10%)
	High‐risk infants (*n* = 28)	Given HBIG	28 (100%)
		Maternal high viral load >1 000 000 iu/mL	23 (82%)
		E‐antigen positive recorded	10 (36%)
		<1.5 kg birthweight	2 (7%)
Infant vaccination documented in High risk *n* = 28 Low risk *n* = 255	Birth dose <24 h	High risk	28 (100%)
		Low risk	239 (94%)
	>4 doses received	High risk	23 (82%)
		Low risk	183 (72%)
	Incomplete course (1–3 doses)	High risk	2 (0.7%)
		Low risk	9 (4%)
Infant serology (*n* = 226)	Serology results available High risk = 28 Low risk = 255	High risk	19 (68%)
		Low risk	207 (81%)
	Age at serology testing (*n* = 226)	≤12 months	3 (1%)
		13–18 months	143 (63%)
		18–24 months	69 (31%)
		>25 months	16 (7%)
		No recorded serology (*n* = 283)	52 (18%)
	Infants with equivocal/positive HBcAb and negative HBs antigen (*n* = 226)	Detected	13 (6%)
		Equivocal	7 (3%)
		Tested <18 months (*n* = 146)	17 (12%)
		Tested 18–24 months (*n* = 69)	3 (4%)
		Repeat testing recorded (negative) (*n* = 20)	5 (25%)
	Infant HBsAb (*n* = 225)	Recorded (*n* = 283)	225 (80%)
		>1000 iu/mL	164 (73%)
		100–1000 iu/mL	44 (20%)
		10–100 iu/mL	14 (6%)
		<10 iu/mL	3 (1%)
	Lost to follow up High risk = 28 Low risk = 255	High risk	9 (32%)
		Low risk	48 (19%)

HBcAb, hepatitis B core antibody; HBsAb, hepatitis B surface antibody; HBV, hepatitis B virus; IQR, interquartile range.

### Maternal tenofovir

Twenty infants (7%) were born to mothers who received TDF in pregnancy; 8 established TDF pre‐conception, and 12 started during the third trimester of pregnancy. The median viral load at initiation was 125 416 376 DNA IU/mL; with one individual having viral load measured at time of delivery (13 300 000 DNA IU/mL). Of those on long‐term TDF, 75% (6/8) showed evidence of viral suppression (HBV DNA <10 IU/mL) on their antenatal bloods; the two that did not meet this criterion had viral loads of 115 DNA IU/mL and 702 000 000 DNA IU/mL. Of those receiving third‐trimester treatment, 33% (4/12) had evidence of viral suppression (reduction of HBV DNA by at least 1–2log_10_).^18^ Of those receiving TDF during pregnancy, 17/20 infants (85%) received HBIG at birth alongside birth dose vaccination.

### Birth dose vaccination

Of the total cohort, 267 (94%) had birth dose vaccination within 24 h of delivery documented. Within the high‐risk group, 28 (100%) had both birth dose vaccination documented and HBIG administration documented. Low‐risk infants had a birth dose vaccination rate of 94% (239), and none received HBIG.

### Infant serology

Overall, 226 (80%) infants had serology testing documented to confirm vertical transmission had not occurred; of those lost to follow up without confirmatory serology testing – 9 were high risk (9/28, 32%) and 48 (48/255, 19%) were low risk (*P* value =0.047). An additional three in the high‐risk group lost to follow up did have initial PCR at 3 months (3/3 negative results), but did not attend subsequent follow up.

Across the cohort, 20 (7%) had initial detectable or equivocal hepatitis B core antibodies (HBcAbs) – the median time of testing for this group was 16 months compared to 17 months for the entire cohort. All those with a detectable or equivocal HBcAb also had a negative HBsAg at the same blood draw evidencing they were remaining maternal antibodies rather than indicating transmission. One quarter, 5/20 (25%), had documented repeat HBcAb at a median of 22 months of age, all of which were negative.

### Infant vaccination

Two hundred and six (73%) had at least four vaccine doses recorded. Of those born prior to the recommendation change in 2017, coverage of ≥4 was 71% (65/91). Of those born after the change, six‐dose coverage was achieved in 66% (124/188) with four‐dose coverage in 73% (138/188).

Of those with recorded serology, 209/226 (92%) had HBsAb >100 IU/mL, the accepted level to ensure a durable protective response has been established – although levels >10 IU/mL are generally accepted to be protective^19^ – and 73% (164/226) achieved HBsAb levels >1000 IU/L. Only 7.5% (17/226) had HBsAb <100 IU/mL, with three of these having HBsAb recorded as <10 IU/mL; two had received three doses of vaccination and one received birth dose with remaining unrecorded; all had negative surface antigen and core antibody when tested (at 16, 24 and 27 months, respectively). Of the subset with HBsAb 10–100 IU/mL, 14 (82%) received at least four vaccine doses. Booster doses are recommended for titres <10 IU/mL or in those who did not receive a full vaccination course.

## Discussion

Prevention of vertical transmission of HBV was universal in those attending, although high‐risk infants were more likely to be lost to follow up than the low‐risk group. The risk stratification is based on maternal/infant factors that increase the risk of vertical transmission of HBV – losing these infants to follow up is therefore of greater concern. Clinic non‐attendance is a barrier to monitoring vaccination, testing serology and providing further health education about ongoing maternal management and future pregnancies. Many factors act as barriers to accessing care, including socio‐economic status, housing insecurity and mobility, disease perception and understanding, particularly where English is not the primary language of the family, and disease‐related stigma within the community.^12^ The post‐partum period is particularly vulnerable for people living with chronic viral infections with high rates of disengagement in care and discontinuation of medication.^20^ People with significant HBV viraemia offered tenofovir in pregnancy may be at increased risk of hepatic flares post‐partum, potentially exacerbated by TDF withdrawal therefore disengagement in care may impact both maternal and infant health.^21^ Evidence‐based targeted interventions to increase engagement and follow‐up of this cohort are required, possibly including improved antenatal education, community‐based follow‐up and third‐sector support.

Tenofovir is recommended for management of pregnant women with HBV where a high viral load (HBV DNA >5.3 _log10_ IU/mL), from the 28th week onwards.^22^ It has been shown to induce maternal viral suppression and therefore prevent vertical transmission^23^; however, the rate of measuring viral load at time of delivery in this cohort was incredibly low. The majority of infants born to women receiving tenofovir still received HBIG at delivery, as they remained in the high‐risk group by UKHSA stratification. Monitoring viral loads after tenofovir is started could demonstrate reduction of infectivity, and be used in re‐stratification of the infants towards the time of delivery. This could assist management planning and reduce the need for HBIG which would have clinical and financial benefits; as well as offering reassurance to families of the reduced likelihood of transmission.

Response to infant HBV vaccination was encouraging with rates of serological protection comparable to national data from 2021 (77% >4 doses, 77% HBsAb >100).^16^ As the recommendation for number of vaccine doses changed within the audited time period as explained, four doses have been treated as a complete course to calculate coverage. Though a significant proportion did not receive a full course, most missed doses did not have an impact on final serology testing with a small minority of 7.5% having HbsAb <100 suggesting many infants form an established response with less than six doses, or the documentation of doses is sometimes incomplete. The transition from the four‐ to six‐dose schedule was driven by alignment with the introduction of routine HBV vaccination at 8, 12, and 16 weeks, whereas acknowledging the need for additional vaccination at birth and 4 weeks for at‐risk infants rather than a need for 6 HBV vaccines to confer protection.^10^

Some infants had positive HBcAb serology, but all were uninfected (HBs antigen negative). Maternal HBcAb transfers placentally and can remain present in infant circulation for up to 24 months.^24^ For those with a positive HBcAb, repeat testing was recommended at 24 months to ensure clearance. This meant an additional burden of sampling for children, appointments for parents and cost of samples and time for the clinic. In low‐risk infants, moving serological testing to 22 months could reduce this burden without negative impact.

## Conclusion

Enhanced engagement for high‐risk infants, delaying serology testing and improving monitoring of viral load in pregnancy, specifically for those receiving TDF, are some of the service improvements being implemented in the Family Hepatitis Clinic. This will improve the rates of completing a vaccination course and having confirmatory serology carried out, thus working towards the WHO's goals of combating viral hepatitis by 2030.

## Ethics Statement

The study was registered with the Imperial College Healthcare Audit Committee, categorised as an audit of routinely collected clinical data, and hence research ethics were not required.
